# Supplementary material for: Resistance of the Wheat Cultivar ‘Renan’ to Septoria Leaf Blotch Explained by a Combination of Strain Specific and Strain Non-Specific QTL Mapped on an Ultra-Dense Genetic Map
Source: Genes (Basel). 2021 Dec 31;13(1):100. doi: 10.3390/genes13010100 (PMC8774678; doi:10.3390/genes13010100)
Supplement: Supplementary file 1 [file genes-13-00100-s001.zip › Langlands-Perry C_supplementary Figures S1-S5.pdf]

SUPPLEMENTARY FIGURE LEGENDS

Figure S1. Histograms representing the phenotypic data distribution for the 2017 datasets. For all of the histograms, the X-axis represents the trait values and the Y-axis represents the number of RILS from the RxCS population, which have the corresponding value for the phenotypic trait. 2017/1 corresponds to the data for Set 1, 2017/2 corresponds to the data for Set 2. The two leftmost columns correspond to the data for RxCS inoculated with I05. The two rightmost columns correspond to the data for RxCS inoculated with I07. Each line corresponds to a phenotypic trait. S14, S20 and S26 are the sporulating area at 14, 20 and 26 days respectively and are expressed in percentages. AUDPCG is the area under the disease progress curve for the green leaf area. AUDPCN is the area under the disease progress curve for the necrotic leaf area. AUDPCS is area under the disease progress curve for the sporulating leaf area. PYC is the pycnidia density. NBS is the number of spores per pycnidiospore. Each trait was studied over two replicates, 1 and 2 for both strains. Columns 1 and 3 correspond to replicate 1, columns 2 and 4 corresponds to replicate 2. The blue dotted line corresponds to the trait value for Renan, the red dotted line corresponds to the trait value for Chinese Spring.

Figure S2. Histograms representing the phenotypic data distribution for the 2018 datasets. For all of the histograms, the X-axis represents the trait values and the Y-axis represents the number of RILS from the RxCS population, which have the corresponding value for the phenotypic trait. The three leftmost columns correspond to the data for RxCS inoculated with I05. The three rightmost columns correspond to the data for RxCS inoculated with I07. Each line corresponds to a phenotypic trait. S14, S20 and S26 are the sporulating area at 14, 20 and 26 days respectively and are expressed in percentages. AUDPCG is the area under the disease progress curve for the green leaf area. AUDPCN is the area under the disease progress curve for the necrotic leaf area. AUDPCS is area under the disease progress curve for the sporulating leaf area. Each trait was studied over three replicates, 1, 2 and 3 for both strains. Columns 1 and 4 correspond to replicate 1, columns 2 and 5 corresponds to replicate 2 and columns 3 and 6 correspond to replicate 3. The blue dotted line corresponds to the trait value for Renan, the red dotted line corresponds to the trait value for Chinese Spring.

Figure S3. Comparison between the RxCS genetic map and the wheat genetic map built by Rimbert *et al.* (2018) [65]. Bars represent chromosomes from 1A to 7D. Green lines link identical SNP between chromosomes. The bar on the left of a pairs corresponds to the map for that chromosome in the Rimbert *et al.* (2018) [65] genetic map. The bar on the right corresponds to the map for that chromosome in the RxCS genetic map.

Figure S4. Comparison between the RxCS genetic map and the RxCS genetic map built by Wang *et al.* (2014) [52]. Bars represent chromosomes from 1A to 7D. Green lines link identical SNP between chromosomes. The bar on the left of a pairs corresponds to the map for that chromosome in the Wang *et al.* (2014) [52] genetic map. The bar on the right corresponds to the map for that chromosome in the RxCS genetic map.

Figure S5. Representations of the RxCS genetic map. Bars represent chromosomes 1A through to 7D. Black lines within bars represent SNP positions. **A**. RxCS genetic map represented with only the mapped SNP originating from the Illumina Infinium iSelect 90K array [52]. **B**. RxCS genetic map represented with all mapped markers from the Illumina Infinium iSelect 90K array [52] and the Breedwheat Affymetrix Axiom 410K array [50,51].

SUPPLEMENTARY FIGURES

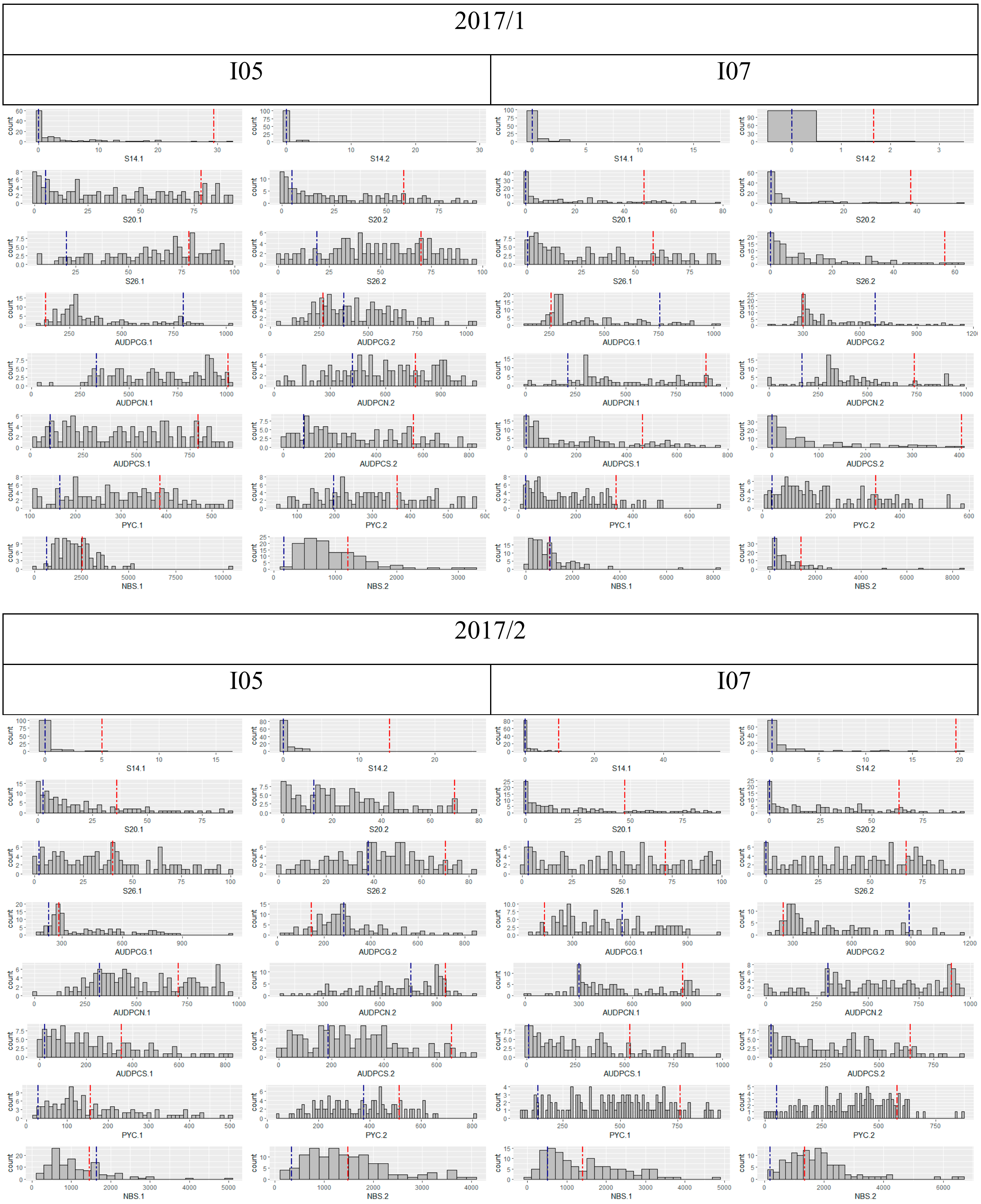

Figure S1. Histograms representing the phenotypic data distribution for the 2017 datasets. For all of the histograms, the X-axis represents the trait values and the Y-axis represents the number of RILS from the RxCS population, which have the corresponding value for the phenotypic trait. 2017/1 corresponds to the data for Set 1, 2017/2 corresponds to the data for Set 2. The two leftmost columns correspond to the data for RxCS inoculated with I05. The two rightmost columns correspond to the data for RxCS inoculated with I07. Each line corresponds to a phenotypic trait. S14, S20 and S26 are the sporulating area at 14, 20 and 26 days respectively

and are expressed in percentages. AUDPCG is the area under the disease progress curve for the green leaf area. AUDPCN is the area under the disease progress curve for the necrotic leaf area. AUDPCS is area under the disease progress curve for the sporulating leaf area. PYC is the pycnidia density. NBS is the number of spores per pycnidiospore. Each trait was studied over two replicates, 1 and 2 for both strains. Columns 1 and 3 correspond to replicate 1, columns 2 and 4 corresponds to replicate 2. The blue dotted line corresponds to the trait value for Renan, the red dotted line corresponds to the trait value for Chinese Spring.

I05	I07

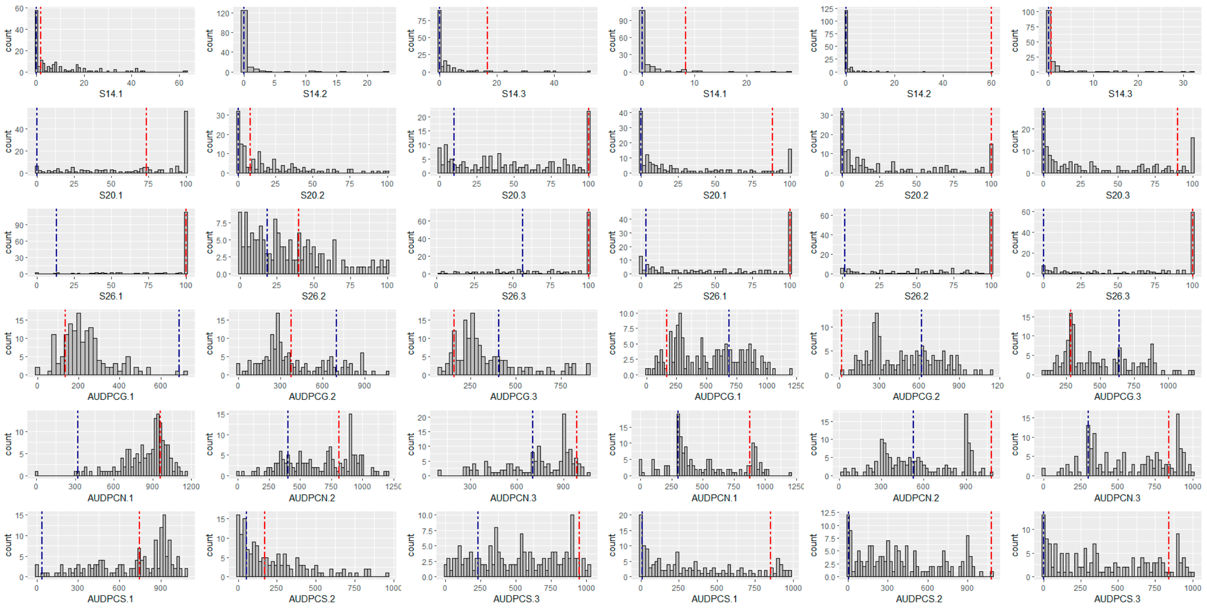

Figure S2. Histograms representing the phenotypic data distribution for the 2018 datasets. For all of the histograms, the X-axis represents the trait values and the Y-axis represents the number of RILS from the RxCS population, which have the corresponding value for the phenotypic trait. The three leftmost columns correspond to the data for RxCS inoculated with I05. The three rightmost columns correspond to the data for RxCS inoculated with I07. Each line corresponds to a phenotypic trait. S14, S20 and S26 are the sporulating area at 14, 20 and 26 days respectively and are expressed in percentages. AUDPCG is the area under the disease progress curve for the green leaf area. AUDPCN is the area under the disease progress curve for the necrotic leaf area. AUDPCS is area under the disease progress curve for the sporulating leaf area. Each trait was studied over three replicates, 1, 2 and 3 for both strains. Columns 1 and 4 correspond to replicate 1, columns 2 and 5 corresponds to replicate 2 and columns 3 and 6 correspond to replicate 3. The blue dotted line corresponds to the trait value for Renan, the red dotted line corresponds to the trait value for Chinese Spring.

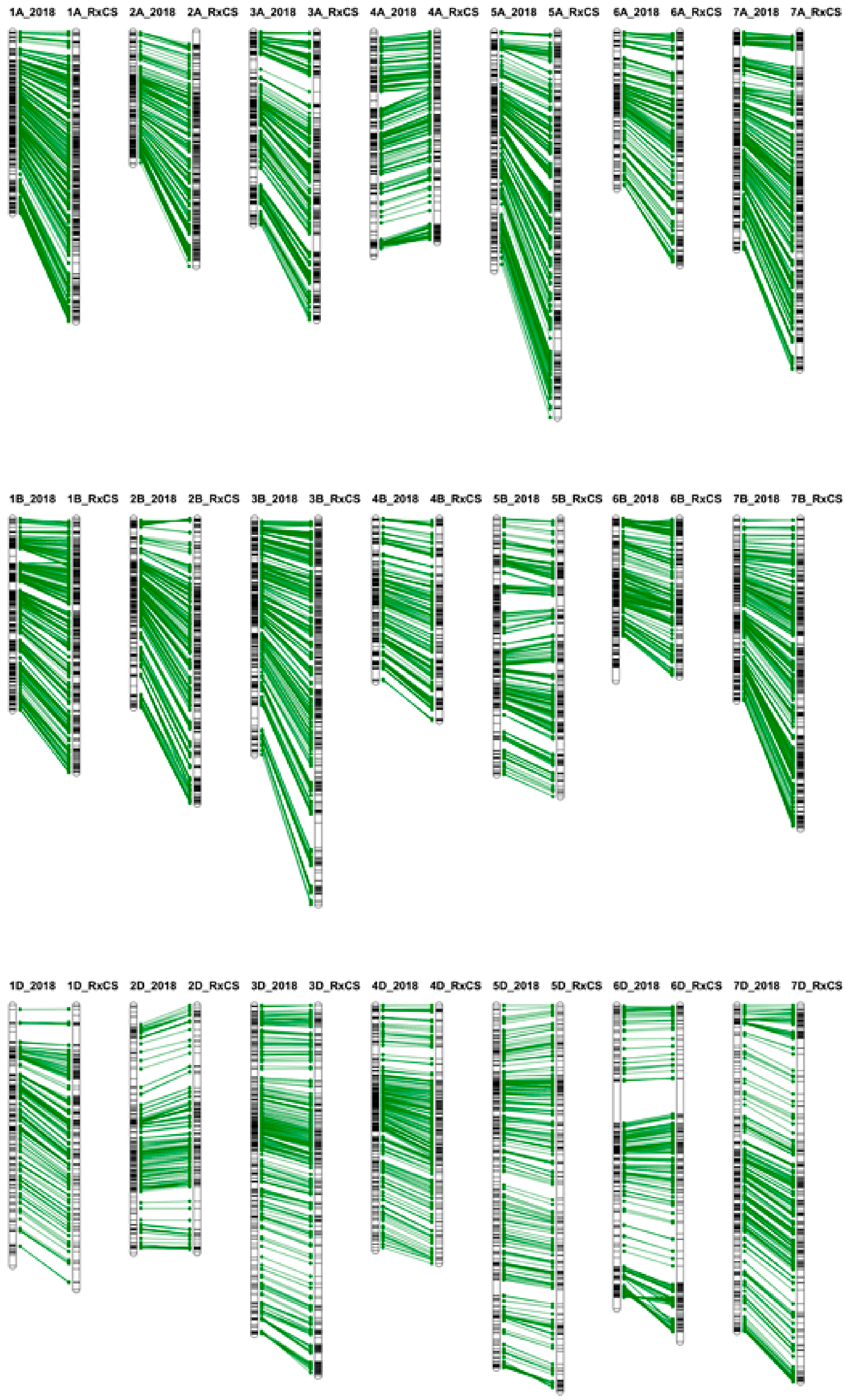

Figure S3. Comparison between the RxCS genetic map and the wheat genetic map built by Rimbert *et al.* (2018) [65]. Bars represent chromosomes from 1A to 7D. Green lines link identical SNP between chromosomes. The bar on the left of a pairs corresponds to the map for that chromosome in the Rimbert *et al.* (2018) [65] genetic map. The bar on the right corresponds to the map for that chromosome in the RxCS genetic map.

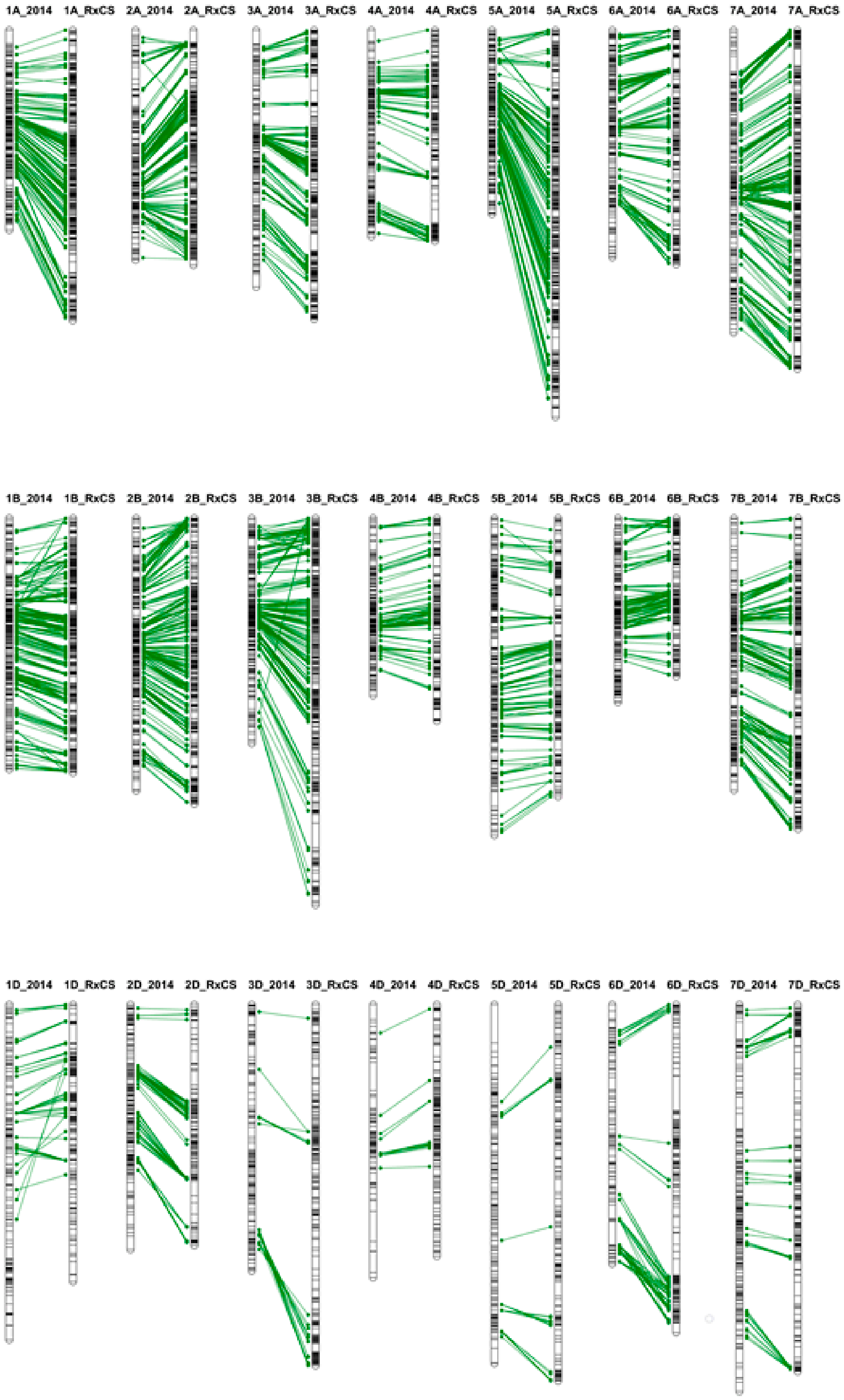

Figure S4. Comparison between the RxCS genetic map and the RxCS genetic map built by Wang *et al.* (2014) [52]. Bars represent chromosomes from 1A to 7D. Green lines link identical SNP between chromosomes. The bar on the left of a pairs corresponds to the map for that chromosome in the Wang *et al.* (2014) [52] genetic map. The bar on the right corresponds to the map for that chromosome in the RxCS genetic map.

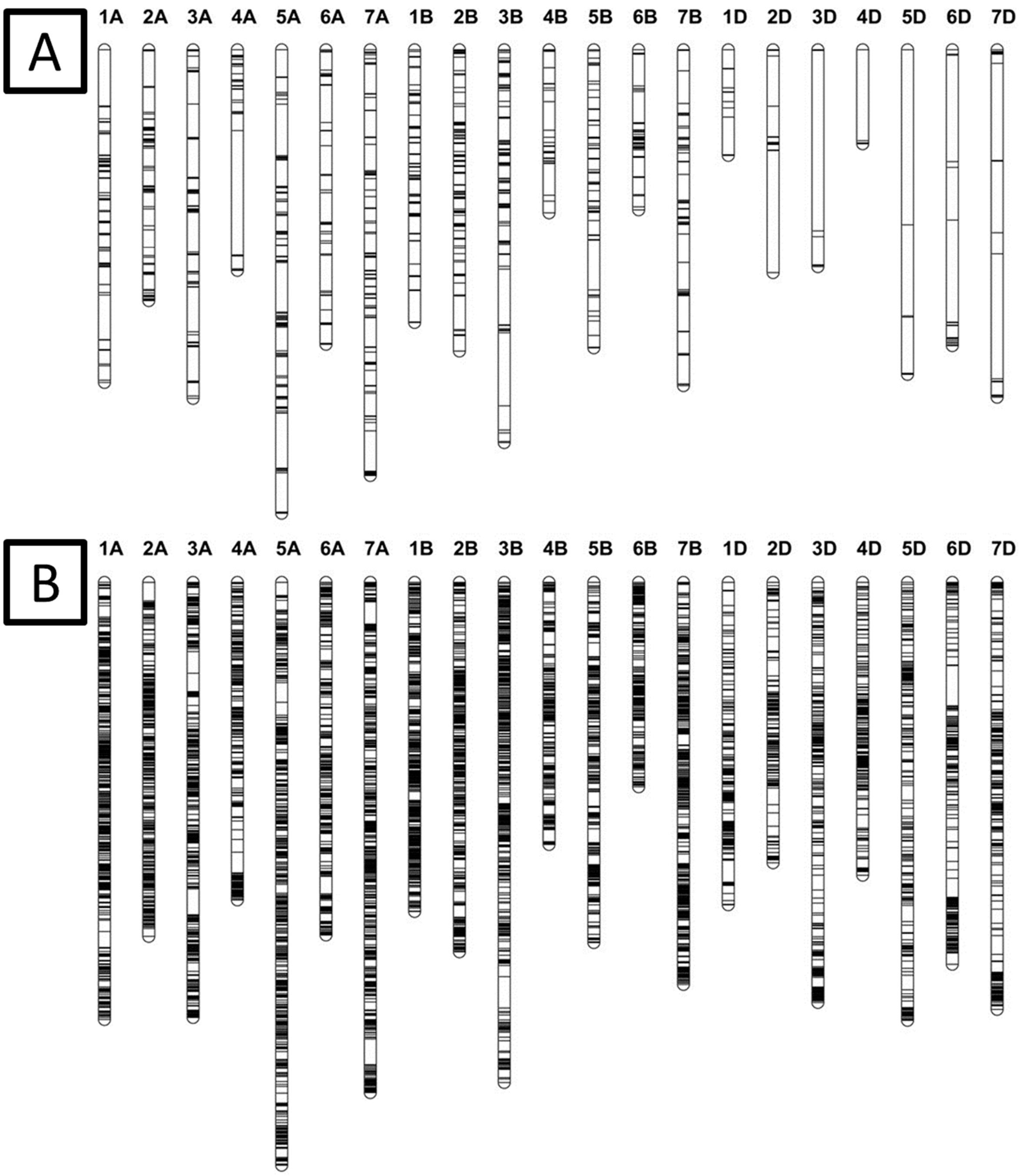

Figure S5. Representations of the RxCS genetic map. Bars represent chromosomes 1A through to 7D. Black lines within bars represent SNP positions. **A**. RxCS genetic map represented with only the mapped SNP originating from the Illumina Infinium iSelect 90K array [52]. **B**. RxCS genetic map represented with all mapped markers from the Illumina Infinium iSelect 90K array [52] and the Breedwheat Affymetrix Axiom 410K array [50,51].
